# TB and HIV Therapeutics: Pharmacology Research Priorities

**DOI:** 10.1155/2012/874083

**Published:** 2012-07-05

**Authors:** Kelly E. Dooley, Peter S. Kim, Sharon D. Williams, Richard Hafner

**Affiliations:** ^1^Johns Hopkins University School of Medicine, 600 N. Wolfe Street, Osler 527, Baltimore, MD 21287, USA; ^2^Division of AIDS, National Institute of Allergy and Infectious Diseases, National Institutes of Health, Bethesda, MD 20892, USA

## Abstract

An unprecedented number of investigational drugs are in the development pipeline for the treatment of tuberculosis. Among patients with tuberculosis, co-infection with HIV is common, and concurrent treatment of tuberculosis and HIV is now the standard of care. To ensure that combinations of anti-tuberculosis drugs and antiretrovirals are safe and are tested at doses most likely to be effective, selected pharmacokinetic studies based on knowledge of their metabolic pathways and their capacity to induce or inhibit metabolizing enzymes of companion drugs must be conducted. Drug interaction studies should be followed up by evaluations in larger populations to evaluate safety and pharmacodynamics more fully. Involving patients with HIV in trials of TB drugs early in development enhances the knowledge gained from the trials and will ensure that promising new tuberculosis treatments are available to patients with HIV as early as possible. In this review, we summarize current and planned pharmacokinetic and drug interaction studies involving investigational and licensed tuberculosis drugs and antiretrovirals and suggest priorities for tuberculosis-HIV pharmacokinetic, pharmacodynamic, and drug-drug interaction studies for the future. Priority studies for children and pregnant women with HIV and tuberculosis co-infection are briefly discussed.

## 1. Introduction

The spread of HIV has fueled the tuberculosis (TB) epidemic, and in less-developed countries, TB is the most common cause of death in HIV-infected individuals, accounting for 22% (350,000) of HIV-related deaths globally [1]. In 2010, 1.1 million of the 8.8 million incident cases of TB worldwide were among people living with HIV [2]. For patients with HIV and TB, there is now strong evidence that treating both diseases concurrently rather than waiting until TB treatment is complete to start antiretroviral (ARV) drugs decreases mortality [3–6]. For this reason, cotreatment is now the standard of care for most patients. Treatment of drug-sensitive TB still requires 6 months of multidrug therapy, but strategies to shorten the treatment duration are being explored and must be tested among patients with and without HIV infection. The coepidemics of TB and HIV have also fostered the global emergence of multidrug-resistant (MDR) TB and extensively drug-resistant (XDR) TB. As such, there is an urgent need for new TB drugs and drug combinations as well as improved approaches to the treatment of TB, particularly in the context of HIV infection. While the current pipeline of new drugs for TB is more robust than it has ever been, advanced planning and active fostering of pharmacokinetic (PK) and pharmacokinetic interaction (PKI) studies with other antimicrobials and ARV drugs are critical to accelerating development and access of new drugs for populations affected by HIV coinfection. These studies are needed to explore the pharmacologic compatibility and tolerability of future combination drug regimens for HIV-TB-coinfected populations. While some PKI studies can be conducted initially among healthy HIV-seronegative volunteers, especially when metabolic drug interactions are expected to result in the need for dose adjustments, it is essential that follow-up studies be conducted among patients with HIV and/or TB so that variability in PK and pharmacokinetic/pharmacodynamic (PK/PD) relationships can be fully explored. 

Many of the potential drug interactions between anti-tuberculosis drugs (both current and in development) and ARV drugs have yet to be evaluated. Current dosing strategies, in these instances, are often based on suboptimal data and/or expert opinion. Furthermore, the need for such data is amplified when applied to special populations, such as, pregnant women and children, as their dosing guidelines are often based on limited data even when HIV coinfection is not a pertinent factor. The first step in addressing these priorities is the design and implementation of early phase clinical trials, including PK and PKI studies that will inform later-phase treatment trials and allow for inclusion of HIV-infected patients taking ARVs in clinical trials of TB regimens. Early consideration and planning of key studies needed in this regard are required to avoid delay in the successful implementation of key treatment strategies for both drug-sensitive and drug-resistant TB in populations living with HIV. 

## 2. Studies of FDA-Approved TB Drugs with ARVs

### 2.1. Rifampin and Rifabutin

Rifamycin antibiotics are an essential part of multidrug regimens for the treatment of drug-sensitive TB. Up to now, no regimen has been identified that effectively treats TB for six months or fewer that does not include a rifamycin throughout treatment [7, 8]. Rifampin (RMP), though, is a promiscuous inducer of drug metabolizing enzymes and drug transporters, and rifamycins reduce concentrations of companion drugs, including ARVs, that are metabolized by cytochrome (CYP) P450 or Phase II enzymes [9]. While efavirenz (EFV-)based antiretroviral therapy (ART) can be used safely together with standard RMP-containing TB regimens in adults, [10, 11] drug-drug interactions between nevirapine (NVP) and RMP are more significant and can potentially lead to clinically significant decreases in NVP plasma concentrations and HIV treatment failure [12]. Also, the effects of RMP on EFV concentrations may depend on a patient's *CYP2B6* metabolizer status; among extensive EFV metabolizers, RMP appears to reduce EFV concentrations, while EFV concentrations are increased among slow EFV metabolizers [13, 14]. Furthermore, there are few options for patients who are resistant to or intolerant of nonnucleoside reverse transcriptase inhibitors (NNRTIs). RMP decreases the plasma concentration of protease inhibitors (PIs) to subtherapeutic levels when the PIs are given at standard doses [15–18]. Coadministration of the PI at higher doses or super-boosting the PI with higher doses of ritonavir (RTV, or r) may result in unacceptably high rates of liver toxicity [18–21]. To complicate things further, risk of toxicity with double-dose or super-boosted PIs varies by patient population (healthy volunteers, children, or adults) and the PI used, as well as other factors, such as preexisting hepatic disease, HIV status, use of companion drugs, such as, isoniazid, and age. 

Although rifabutin (RBT) is a less potent inducer of cytochrome P450 enzymes and is less likely to reduce concentrations of coadministered PIs, it is not yet widely available in developing countries, though access is rapidly expanding [22]. Further, RBT (and its main metabolite) are substrates of CYP3A, [23] leading to bidirectional drug interactions with PIs. For example, giving RTV, a potent inhibitor of CYP3A, together with RBT increases concentrations of RBT and its 25-O-desacetylrifabutin metabolite substantially [24]. This is concerning because rifabutin-induced uveitis is thought to be dose dependent. Though it is clear that RBT dose must be reduced when RBT is given together with RTV-boosted PIs, the optimal dose and dosing frequency has not been determined experimentally. Reducing the dose from 300 mg daily (standard dose) to 150 mg thrice weekly so that RBT can be given together with PIs may be associated with subtherapeutic levels of RBT, [7] especially in HIV-infected persons, leading to increased risk of treatment failure and development of drug resistance [8]. Reducing the dose to 150 mg but giving it once daily with RTV-boosted PIs results in therapeutic or supratherapeutic RBT concentrations, reducing the risk of TB treatment failure, but the associated risk of toxicities, such as, uveitis or neutropenia related to elevated parent drug and metabolite exposures is unknown. 

Better strategies for cotreating HIV and TB in patients with NNRTI resistance/intolerance are urgently needed. There are 3 potential strategies that can address these issues with currently available anti-TB drug regimens.

1. *Optimize the Dose of RBT and Use PI-Based HAART*. This approach requires determination of the best dose of RBT when used in combination with various PI-based ARV regimens. The French Agence Nationale de Recherche sur le SIDA (ANRS) recently sponsored a study to evaluate the pharmacokinetics of RBT combined with antiretroviral therapy (EFV, NVP, or lopinavir (LPV)/r) in patients with TB-HIV coinfection in South Africa (RBT 450 mg once daily (QD) versus RBT 600 mg QD with EFV; RBT 300 mg QD versus RBT 450 mg QD with NVP; RBT 150 mg three times per week (TPW) versus RBT 150 mg QD with LPV/r) (NCT00640887, results awaiting publication) and is currently sponsoring a study of RBT 150 mg TPW versus QD and LPV/r in Vietnam (NCT00651066). Another current study being performed at the Harriet Shezi Children's Clinic in South Africa will evaluate the dosing, safety, and pharmacokinetic profile of RBT in children receiving concomitant treatment with LPV/r and RBT (NCT01259219). Lastly, ACTG 5290 is a trial sponsored by the US National Institute of Allergy and Infectious Diseases (NIAID) and performed by the AIDS Clinical Trials Group (ACTG) that will evaluate RBT 150 mg QD when given with standard dose LPV/r with or without raltegravir (RAL) versus a RMP-based TB treatment with double-dose LPV/r (all as part of multidrug TB and HIV treatment) among HIV-infected participants with TB. In this study, the PK of RBT will be evaluated, and safety and treatment efficacy data will be collected. Following an initial stage to obtain PK and safety data, the doses of RBT and LPV/r may be adjusted prior to proceeding to the second stage of the study.
2. *Keep RMP and Give PI-Based HAART but Increase the Dose of the PI or Its Pharmacoenhancer*. This approach requires determination of the optimal dose of the PI of interest along with its paired pharmacoenhancer (usually RTV). In a small group of patients with HIV in South Africa taking LPV/r at standard doses, a gradual stepwise increase in LPV/r dose from 400/100 mg to 800/200 mg when RMP was added was relatively well tolerated with less hepatotoxicity than has been seen in studies in healthy HIV-seronegative volunteers, but this strategy remains to be tested in a larger cohort [25]. The HIV Netherlands Australia Thailand Research Collaboration (HIV-NAT) is currently evaluating the PK and safety of two different doses of LPV/r in HIV-TB-coinfected patients receiving RMP-containing antituberculosis therapy (NCT01138202). Another study by the Oswaldo Cruz Foundation and Abbot Pharmaceuticals is evaluating the pharmacological interaction of LPV/r and RMP (A06-295) (NCT00771498). Lastly, as noted above, ACTG 5290 will also address this question with an arm that tests the antiviral efficacy and safety of double-dose LPV/r + RMP. Besides these studies, trials to determine the optimal dose of other PIs such as darunavir (DRV) are needed. No PKI studies involving cobicistat, a novel pharmacoenhancer, with a coadministered PI and RMP have been conducted.
3. *Keep RMP and Use an Integrase Inhibitor instead of or in Combination with a PI*. This strategy requires evaluation of the antiviral effectiveness of this approach among coinfected patients. RAL is metabolized by UDP-glucuronosyltransferase (UGT) 1A1, an enzyme that is induced by rifamycins. Initial PK studies among healthy volunteers demonstrated that giving RAL at twice the standard dose (800 mg twice daily) with RFP resulted in similar overall RAL exposures as RAL given at 400 mg twice daily alone, but trough concentrations were diminished [26]. Though low trough concentrations of RAL when the drug is given once daily are associated with virologic failure [27, 28], the clinical significance of reductions in trough concentrations when RAL is given twice daily is unknown. In the REFLATE trial, ANRS is evaluating higher doses of RAL together with an NRTI backbone among patients taking RMP-based TB treatment (NCT00822315). Clinically relevant results, including virologic suppression and immunologic response, will help guide recommendations for this possible combination. One study evaluating the effect of thrice weekly RMP on RAL concentrations is being planned, and this will contribute to our knowledge about the relationship between RMP dosing frequency and induction of metabolizing enzymes (NCT01424826). Intermittent dosing of RMP, though, is not recommended for patients with HIV and low CD4+ lymphocyte counts. A Phase 1 study evaluating the effect of RMP on dolutegravir, a new-generation integrase inhibitor, in healthy HIV-seronegative volunteers has shown promising results, but TB-HIV regimens including RMP and dolutegravir must be tested in patients to ensure HIV treatment efficacy with this strategy (NCT01231542).

Three studies evaluating the treatment-shortening potential of higher-dose RMP are in development, sponsored by the Division of Microbiology and Infectious Diseases (DMID) at NIH and the European and Developing Countries Clinical Trials Partnership (EDCTP) (HighRIF NCT00760149; HR1 NCT01392911; HIRIF NCT01408914). Should higher-dose RMP prove effective for shortening the duration of treatment needed for drug-sensitive TB, then evaluation of the relationship between RMP dose and induction of the enzymes that play a major role in biotransformation of ARVs, such as CYP3A, CYP2B6, and UGT1A1 should be explored.

### 2.2. Rifapentine

Approved by the Food and Drug Administration (FDA) for the treatment of TB in 1998, rifapentine (RPT) is the most recently licensed TB drug and is the newest of the rifamycins. In mouse studies, RPT has potent activity, and substitution of RMP with RPT allows for shortening of treatment duration for drug-sensitive TB when given as part of a multidrug regimen. However, the currently approved dosing regimen (600 mg twice weekly during the intensive phase of TB treatment and 600 mg once weekly during the continuation phase of TB treatment) has been associated with treatment failure and development of rifamycin resistance in some populations, including those with advanced HIV [29]. Some feel that intermittent dosing of isoniazid, which has a short half life, and RPT, which has a longer half life, results in “PK mismatch,” leading to periods of effective monotherapy which promote the emergence of rifamycin resistance, and this issue remains hotly debated and is of considerable clinical importance [30–32]. One multicenter randomized controlled trial (RIFAQUIN) is evaluating RPT given together with moxifloxacin, two drugs with similar half lives, once or twice weekly during the continuation phase of TB treatment (http://www.edctp.org/). Total treatment duration will be four months (two months of daily moxifloxacin, rifampin, pyrazinamide, and ethambutol followed by two months of once- or twice-weekly moxifloxacin and RPT). 

Three studies to determine the optimal daily dose of RPT as part of multidrug treatment for TB are currently enrolling, including a study evaluating RPT doses up to 20 mg/kg daily (TBTC Study 29X) given together with standard companion drugs (isoniazid (INH), pyrazinamide, and ethambutol), one study evaluating daily RPT doses of 450 and 600 mg (with standard companion drugs), and another testing substitution of moxifloxacin for INH and substitution of RPT at a dose of 300–450 mg daily for RMP, (NCT00728507, NCT00814671, NCT00694629). An ACTG study of different dosing strategies to maximize drug exposure, including divided dosing and different meal types is also in the planning stages. Thus, the optimal dosing frequency, dose, and companion drugs for RPT for the treatment of active TB are under active investigation.

RPT induces P450 metabolizing enzymes and Phase II enzymes, similar to its analogue, RMP. The risk of drug interactions when RPT is given together with ARVs that are P450 substrates or are metabolized by Phase II enzymes is high. A study of the effect of daily versus once weekly RPT on RAL in healthy individuals by the Tuberculosis Clinical Trials Consortium (TBTC) has recently been completed, and results are expected soon (NCT00809718). In this study, as well as the RIFAQUIN study, the relationship between RPT dosing intermittency and the induction of the Phase II enzymes that metabolize RAL and moxifloxacin, respectively, is being explored. Other PKI studies with ARVs that require consideration are studies of daily RPT once the optimized dose is determined plus EFV, NVP, and key PIs, such as, RTV-boosted LPV and DRV. A recent study evaluating CYP3A induction by RPT using oral midazolam as a probe drug revealed that RPT may have greater induction effects than RMP at clinically relevant doses, so recommendations for dose adjustments based on RMP drug-drug interaction studies may not be readily extrapolated to RPT [33]. ACTG 5279 is a trial evaluating the efficacy of one month of daily RPT (dosed at 450 or 600 mg, depending on weight) + INH versus 9 months of daily INH for the treatment of latent TB infection (LTBI) in HIV-infected individuals (NCT01404312). This trial includes an evaluation of the drug-drug interaction between RPT and NNRTIs.

A recent, large, randomized clinical trial showed that directly observed therapy with RPT and INH, each given once weekly for twelve weeks, was noninferior to INH given daily for nine months for the treatment of LTBI [34]. The relationship between dosing frequency and induction of P450 enzymes by RPT has not been tested, so PKI studies involving RPT dosed weekly and key ARVs are warranted to ensure that this 12-week LTBI treatment is safe for patients with HIV taking ARVs and does not compromise the efficacy of their HIV treatment.

### 2.3. Isoniazid

Lastly, given the growing practice of treating LTBI in HIV-infected individuals with INH in developing countries, the effects of INH on the kinetics of companion ARVs, namely, EFV and LPV/r, should be evaluated. INH is a potent inhibitor of CYP2C19 and CYP3A metabolizing enzymes [35], and while the inducing effects of RMP overpower the inhibitory effects of INH when used together, the effects of INH in the absence of RMP have not been well delineated.

## 3. HIV-TB PKI Studies of Select TB Drugs in the Pipeline

The current pipeline of new TB drugs represents the most robust portfolio of new drugs in development in the history of TB research. Many of these drugs have advanced to Phase 2 studies and offer the potential for increasingly efficacious TB regimens for both drug-sensitive and drug-resistant disease. Though they represent the future of TB treatment strategies, some of them have or are expected to have clinically significant interactions with current TB drugs and HIV treatment regimens. Thoughtfully selected, appropriately timed PKI studies are essential to ensure that these new regimens will benefit HIV-infected populations as well as those uninfected with HIV.

### 3.1. Bedaquiline (Formerly TMC-207) (Janssen for MDR TB, TB Alliance for Drug-Sensitive TB)

Bedaquiline is a first-in-class diarylquinoline that inhibits bacterial ATP synthase and is proven to have potent activity against MDR TB in randomized, placebo-controlled clinical trials [36, 37]. Bedaquiline has a long terminal half life of over five months, complicating PKI studies and is a CYP3A substrate with moderate to high-risk of drug-drug interactions with CYP3A4 inducers or inhibitors. Its concentrations are reduced by about 50% when given with RMP or RPT, [38] and the effect of RBT on bedaquiline PK is being evaluated in a currently enrolling trial (NCT01341184). Several studies evaluating drug-drug interactions with high-priority ARV drugs have already been completed. Results from a study evaluating the safety and PKI of single-dose bedaquiline with steady-state EFV revealed a good safety profile with bedaquiline concentrations only modestly reduced [39]. Analyses to estimate steady state concentrations of bedaquiline and its M2 metabolite using nonlinear mixed effects modeling is in progress to ensure that accumulation of bedaquiline's M2 metabolite is not a concern [40]. Coadministration of NVP with bedaquiline was well tolerated among patients with HIV on NVP-based ART and did not influence the bedaquiline area under the time-concentration curve (AUC) and reduced the maximum concentration (Cmax) by only 20% [41]. Evaluation of the effect of LPV/r on single doses (400 mg) of bedaquiline revealed that coadministration had no effect on the Cmax of bedaquiline, and the AUC was increased by only 22% [42]. The PK parameter that correlates best with treatment response and PK targets for bedaquiline have not been determined, so it is unclear what reductions in bedaquiline concentrations would be clinically relevant. If bedaquiline is tested in RMP-containing regimens for drug-sensitive TB, the combined inductive effects of RMP and EFV would need to be evaluated before enrolling participants taking EFV-based ART. Higher doses of bedaquiline in this setting could only be used if metabolite concentrations were in an acceptable range.

Lastly, given that the PKI studies to date were conducted in healthy volunteers receiving single doses of bedaquiline and that steady-state concentrations of bedaquiline are difficult to predict from single-dose data given the triphasic elimination of the drug and its exceedingly long terminal half-life, longer term studies involving multiple doses of the drug in patients with TB and HIV will be essential for furthering our understanding of the PK and pharmacodynamics (PDs) associated with this drug and the effects of ARVs on bedaquiline PK.

### 3.2. Nitroimidazoles-PA-824 (TB Alliance) and Delamanid (Formerly OPC-67683) (Otsuka)

PA-824 (TB Alliance) and delamanid (Otsuka) are drugs in the nitroimidazole class with activity against both metabolically active and nonreplicating *M. tuberculosis,* including drug-sensitive and drug-resistant strains [43]. Delamanid is not metabolized by human liver microsomes and does not induce P450 enzymes, so it poses relatively low metabolic drug interaction risk. PA-824 is a weak competitive inhibitor of CYP 3A, 2C8, 2C9, and 2C19, and it is 20% metabolized by 3A4, so its drug interaction liability is, likewise, low. A study evaluating the effects of EFV and LPV/r on PA-824 concentrations (and vice versa) is currently being planned by the ACTG in collaboration with the TB Alliance. In the ACTG study, evaluation of the effects of RMP on PA-824 PK will also be assessed. Given the potential importance of these drugs in future TB drug combinations, additional studies evaluating drug interactions with other new TB drugs or ARVs may be necessary if overlapping toxicities or interactions affecting absorption are suspected. For example, a combination of PA-824, moxifloxacin, and pyrazinamide showed superior activity to standard TB treatment in mouse models and in a two-week early bactericidal activity (EBA) study in humans [44, 45]. Though no metabolic drug interaction is expected based on the metabolic pathways of the three drugs, a drug interaction study evaluating the combined effect of PA-824 and moxifloxacin on the QT interval is under development. 

### 3.3. SQ109 (Sequella)

SQ109 is a [1,2]-ethylenediamine-based drug with structural similarities to ethambutol (EMB) but is ten times more active than EMB in preclinical models [46]. The mechanism of action involves disruption of cell wall assembly but is distinct from that of ethambutol [47]. *In vitro* studies suggest synergy between SQ109 and RMP or INH, [48] and EDCTP-supported clinical trials evaluating SQ109 alone and in combination with other TB drugs are underway (NCT01218217). SQ109 has a terminal half life of 40–50 hours, (Personal Communication, Gary Horwith, Sequella) and *in vitro* experiments suggest that SQ109 is metabolized by CYP2D6 and 2C19, [49] so there is moderate drug-drug interaction risk for this compound when given together with drugs that induce or inhibit those enzymes. A PKI study involving SQ109 and RMP has not, to our knowledge, been done and is of the highest priority especially if synergy between the two drugs is expected from preclinical models and will be tested clinically. Given its potential use for both drug-sensitive and drug-resistant TB disease, a PKI study with SQ109 and a PI/r combination represents an important consideration once a dose going forward is established, especially given that drug interactions with RTV can be highly unpredictable. Additionally, given the frequent use of fluconazole in the HIV population and fluconazole's strong inhibition of 2C19, a PKI study of SQ109 with fluconazole is likely to provide clinically significant information if concentration-dependent toxicities are expected or seen in Phase 2 studies. 

### 3.4. Oxazolidinones–Sutezolid (Formerly PNU-100480) (Pfizer) and AZD5847 (AstraZeneca)

Sutezolid (formerly PNU-100480, Pfizer) and AZD5847 (AstraZeneca) are new oxazolidinones in phase 2 development for TB [50]. Sutezolid is largely metabolized by flavin monooxygenases to sulfoxide and sulfone derivatives [51]. The sulfoxide metabolite is present in plasma at five to seven times the concentration of the parent drug and may contribute significantly to the drug's activity. CYP3A4 is responsible for about 30% of sutezolid's metabolism. Neither the parent drug nor the metabolites appear to be inhibitors or inducers of CYP3A4. The dose going forward is not known for sutezolid or for The EBA study of AZD5847 is planned for the future, and results from the EBA study of sutezolid are expected soon (NCT01225640, NCT01516203). Once the doses to be tested in later-phase studies are determined, then drug interaction studies can be considered. However, these would need to be carefully designed and take into consideration the fact that sutezolid's major metabolite circulates at higher concentration and may be more active than the parent drug. 

## 4. Special Populations

Though the large burden of TB among pediatric populations is widely recognized, children represent a historically understudied population in TB research. Given that efficacy trials in adult TB patients depend on production and culture of sputum samples during treatment and young children cannot produce sputum samples for testing, dosing recommendations for TB drugs for children are generally based on PK studies rather than efficacy trials. New compounds that have demonstrated efficacy for which a dose has been selected in adults should immediately be tested in children, beginning in adolescents, and then proceeding to progressively younger groups of children. The importance of this is illustrated by the example of INH and RMP. When given to children at the same mg/kg dose as adults, concentrations are much lower, and children were treated with suboptimal doses of these drugs for decades. Only recently were the doses recommended by the World Health Organization increased to reflect these age-related differences in drug disposition [52]. A pediatric dose finding study of bedaquiline among children with MDR-TB is under development by the NIAID-funded International Maternal Pediatric Adolescent AIDS Clinical Trials Group (IMPAACT) network with support from Janssen. Similar dose finding studies are required for all of the new TB drugs in the pipeline as well as many currently existing TB drugs. Development of formulations that can be used in young children is a high priority for all promising investigational TB drugs.

The magnitude and variability of drug interactions may be expected to be different in pediatric populations than in adults, as expression of key metabolizing enzymes changes as children develop [53], and responses to drugs that inhibit or induce metabolizing enzymes may also vary with age. Therefore, dose adjustments for ARVs when taken together with rifamycins do not necessarily follow from adult recommendations and should be tested specifically in children to ensure adequate drug exposures. For example, while double-dose ritonavir-boosted LPV may result in adequate LPV concentrations in adults taking RMP, the same is not true among children [25, 54]. Similarly, NVP concentrations in young children are substantially reduced by RMP coadministration [55]. Determining the optimal doses of RTV and LPV to give children who are also taking RMP for TB remains a high priority, particularly among very young children for whom EFV dosing recommendations have not been established and is under active investigation [54, 56, 57]. RBT is not available in a pediatric formulation, so substitution of RMP with RBT is not currently an option.

HIV/TB drug interactions in pregnant women and the impact of these interactions on prevention of mother-to-child transmission also deserve attention. Up to now, there have been no published data on the combined effects of pregnancy and RMP on ARV concentrations and efficacy of HIV treatment. For women with HIV and TB who cannot receive EFV because of potential teratogenic effects early in pregnancy and for whom NVP is contraindicated because of CD4 count higher than 250 cells/mm^3^, options are limited. The safety, PK, and efficacy of higher dose LPV/r or RAL when given with RMP-containing TB treatment have not been tested in pregnant women. 

## 5. Conclusion

The coepidemics of TB and HIV represent a deadly marriage of global significance. Advances in the treatment of drug-sensitive and drug-resistant TB, though, are likely in the near future given the increased number of drugs in the development pipeline and promising results in preclinical and early clinical studies of regimens involving existing and investigational drugs. To ensure that patients with HIV can fully benefit from new and currently available TB regimens, studies evaluating the safety, pharmacokinetics, and efficacy of coadministered antiretrovirals and antituberculosis drugs must be undertaken, particularly when metabolic drug interactions or overlapping toxicities are likely (Table 1). Consideration and advanced planning of the most pertinent studies should begin early in the course of drug development with guidance from preclinical studies and should be done before phase IIb trials of multidrug TB regimens. While Phase I studies using crossover designs may be employed for drugs with shorter half lives, for drugs with longer half lives or time-dependent kinetics, nesting PKI studies in Phase IIa treatment trials will be the most informative strategy. For drugs whose concentrations are highly dependent on environmental and host factors, including, genetics, PKI studies must be conducted in the relevant populations, including in high-burden settings, rather than extrapolating results from trials conducted among a small subset of participants, such as, healthy volunteers. Advocacy together with funding support from industry, government, and public-private sources will be needed to ensure that PKI involving investigational TB drugs and relevant ARVs is conducted, particularly when interactions are predicted and dose adjustment strategies must be explored. PK studies to find the age-appropriate dose of investigational agents for children should be conducted as soon as a dose going forward in adults is determined, with special attention to drug formulation. Finally, sparse PK sampling in all large clinical trials of new TB drugs or regimens will help define the PK/PD parameters that correlate best with treatment response and determine PK targets to ensure optimized dosing. Concurrent treatment of HIV and TB saves lives, and careful assessment of the pharmacology of coadministered antiretrovirals and antituberculosis drugs will help ensure that new or improved TB regimens will benefit those patients who need them most.

## Figures and Tables

**Table 1:** High-priority TB-HIV PK or PK/PD studies.

FDA approved TB drugs with ARVs	
Rifampicin (RMP)	
(i) Evaluation of double-dose LPV/r (800/200 twice daily) among HIV/TB-coinfected adults taking RMP-containing TB treatment with focus on HIV viral suppression and hepatotoxicity	
(ii) Evaluation of super-boosted LPV (1: 1 LPV/r ratio with weight-based dosing) among HIV/TB-coinfected children receiving RMP-containing TB treatment with focus on LPV PK and HIV viral suppression	
(iii) Evaluation of EFV at 600 mg versus 800 mg daily among patients with HIV/TB-coinfection taking RMP-containing TB treatment who weigh more than 50 kg	
(iv) Evaluation of EFV or higher dose LPV/r among pregnant women with HIV/TB-coinfection taking RMP-containing TB treatment	
(v) Evaluation of double-dose RAL (800 mg twice daily) among HIV/TB-coinfected patients taking RMP-containing TB treatment	
(vi) Determination of dose of DRV/r likely to achieve target DRV concentrations among subjects taking RMP	
(vii) Evaluation of higher dose dolutegravir (50 mg twice daily) among HIV/TB-coinfected patients taking RMP-containing TB treatment	

Rifapentine (RPT)	
(i) Drug interaction studies involving RPT at the optimized dose for TB treatment and key HIV drugs, namely, EFV, NVP, and ritonavir-boosted PIs	
(ii) Drug interaction studies with weekly RPT used for LTBI treatment and key ARVs including EFV, NVP, and ritonavir-boosted PIs among patients with HIV receiving ART	
(iii) Dose-finding PK study in infants and pediatric formulations	

Rifabutin (RBT)	
(i) Evaluation of RBT at a dose of 150 mg daily among HIV-TB-coinfected patients taking LPV/r-based ART with a focus on RBT-related toxicities and HIV viral load suppression	
(ii) RBT formulations for children	

Isoniazid (INH)	
(i) Studies of the effects of INH (alone) as treatment for LTBI on the kinetics of EFV and LPV/r	

Select Tb drugs in the pipeline	

AZD5847	
(i) PKI studies as appropriate once the dose to be tested in later-phase studies is determined and information regarding its metabolism and capacity to induce or inhibit P450 enzymes is publicly available	

Bedaquiline (TMC207)	
(i) PK/PD studies among patients with TB/HIV coinfection taking bedaquiline-containing TB treatment and ART that includes EFV or NVP (multiple dose study)	
(ii) Dose-finding PK study in children with MDR-TB	
(iii) PKI with combined use of RMP and EFV with bedaquiline	

Delamanid (OPC-67683)	
(i) No specific drug interaction studies currently recommended given low risk of metabolic drug interactions	

PA-824	
(i) Drug interaction studies with RMP, EFV, and LPV/r with necessity of further studies to be determined by results of these trials of PA-824 given with a potent inducer (RMP) or potent inhibitor (ritonavir)	

Sutezolid (PNU-100480)	
(i) Drug interaction study with RMP and perhaps key ARVs once dose to be tested in later-phase studies is determined, with measurement of parent drug and active metabolites	
(ii) Dose-finding PK study in children	

SQ109	
(i) PKI study with RMP once the dose of SQ-109 to be tested in later-phase studies is determined	
